# Izumo1 and Juno: the evolutionary origins and coevolution of essential sperm–egg binding partners

**DOI:** 10.1098/rsos.150296

**Published:** 2015-12-16

**Authors:** Phil Grayson

**Affiliations:** Organismic and Evolutionary Biology, Harvard University, 26 Oxford Street, Cambridge, MA 02138, USA

**Keywords:** Izumo1 and Izumo family, Juno and folate receptor family, reproduction and fertilization, positive selection, gene family evolution, coevolution

## Abstract

Reproductive proteins are among the most rapidly evolving classes of proteins. For a subset of these, rapid evolution is driven by positive Darwinian selection despite vital, well-conserved, reproductive functions. Izumo1 is the only essential sperm–egg fusion protein currently known on mammalian sperm, and its egg receptor (Juno; formerly Folr4) was recently discovered. Male knockout mice for Izumo1 and female knockout mice for Juno are both healthy but sterile. Here, both sperm–egg binding proteins are shown to be evolving under positive selection. Within mammals, coevolution of Izumo1 and Juno is also uncovered, suggesting that similar forces have shaped the evolutionary histories of these binding partners within Mammalia. Additionally, genomic analyses reveal an ancient origin for the Izumo gene family, initially reported as conserved exclusively in mammals. Newly identified Izumo1 orthologues could serve reproductive functions in birds, fish and reptiles. Surprisingly, these same analyses support Juno's presence in mammals alone, suggesting a recent mammalian-specific duplication and neofunctionalization of the ancestral folate receptor. Despite the indispensability of their reproductive interaction, and their apparent coevolution within Mammalia, this binding pair arose through strikingly different evolutionary forces.

## 1. Introduction

In sexually reproducing species, fertilization occurs through the recognition and fusion of haploid sperm and egg to produce a diploid zygote. Although the processes involved in this essential reproductive stage have been well studied, the molecular basis of mammalian sperm–egg fusion was largely unknown until recently. The first essential sperm–egg fusion protein to be discovered on mammalian sperm was Izumo1. An Izumo1 knockout results in male mice that are completely sterile, but otherwise healthy, while female mice exhibit a normal phenotype [1]. Recently, the egg receptor for mammalian Izumo1 was discovered to be folate receptor 4 (Folr4), which was renamed Juno (or Izumo1R) [2]. Juno knockouts result in a phenotype opposite to Izumo1 knockouts: healthy, yet sterile female mice, and phenotypically normal males [2]. Izumo1 and Juno are the first essential sperm–egg binding pair identified in any species, and their conserved binding ability has been demonstrated in numerous mammals [1–3]. Additional mammalian egg proteins, including CD9 and CD81, are also known to be essential for sperm–egg fusion [4]. Currently, it is unclear whether these egg proteins interact directly with a binding partner on the sperm, and their precise roles in sperm–egg fusion have yet to be determined [4–7].

Comparative genomic studies have identified reproductive proteins as one of the most rapidly evolving gene-classes across diverse taxa [8]. *A priori*, one might expect positively selected reproductive genes to play secondary roles in reproductive success while proteins central to fertilization remain generally conserved, but positive selection has been identified in many classes of reproductive proteins including those functioning in sperm competition, sperm–egg binding and spermatogenesis [7,9–12]. Although Izumo1 has an indispensable role in fertilization, it too has been identified as evolving rapidly through positive selection in both large exploratory studies and in-depth targeted analyses [7,11,13].

Izumo1 paralogues have also been discovered, resulting in a four-member Izumo family in mammals [14]. Izumo1, Izumo2 and Izumo3 are expressed exclusively in the testes, while Izumo4 lacks a transmembrane domain and is present throughout the body [14]. Molecular evolutionary study revealed that Izumo1 is evolving under positive selection across Mammalia, driven by rapid evolution within the laurasiatherian mammals, while Izumo2 is positively selected within the Primates, and Izumo4 is evolving rapidly in the Glires (Rodentia and Lagomorpha), probably due to a relaxation of selective constraint [13].

Juno, previously Folr4, is also a member of a four-gene family. The mammalian folate receptor family consists of Folr1, Folr2 and Folr4 across Mammalia, and Folr3 within Primates [15–18]. Folr1–3 are known to bind folate by a newly described mechanism, and the heightened expression of Folr1 and Folr2 in disease-causing cells in cancers and inflammatory diseases has led to extensive research into folate receptor-targeted therapies [19–22]. Human and mouse Juno differ from the other family members at a number of essential folate-binding sites and are indeed unable to bind folate [2]. In mice, Folr1 and Folr2 do not interact with Izumo1; similarly, Izumo3 and Izumo4 do not interact with Juno [2].

Molecular evolutionary analyses of gene families not only provide insight into the history of the genes, but can also identify targets for future functional experiments [23]. No such study has been conducted for the folate receptor family. Here, I describe the evolutionary history of the only essential sperm–egg fusion pair currently known, Izumo1–Juno, in the context of their respective gene families. I examine the evolutionary history of the folate receptor family in vertebrates for the first time and conclude that Juno arose through a recent, mammalian-specific duplication and neofunctionalization of an ancestral folate receptor gene. I uncover an ancient origin of the Izumo gene family and Izumo1, which has only been previously described and studied in mammals. These newly discovered Izumo1 orthologues could serve similar reproductive functions in fish, birds and reptiles. I identify positive selection driving the evolution of Juno within mammals and compare these signatures of selection to those previously reported for Izumo1 [13]. Finally, I investigate coevolution between Izumo1 and Juno within Mammalia and identify lineage-specific signals of coordinated evolution between these binding partners. I conclude that Izumo1 and Juno have distinct temporal origins, but coevolve under similar selective pressures within the mammals.

## 2. Methods and results

### 2.1 Folate receptor family evolution

Homologous sequences for mammalian Folr1, Folr2, Folr3 and Folr4 were identified from NCBI and Ensembl. These sequences were evaluated through a suite of techniques: reciprocal BLAST searches on NCBI [24,25], visual inspection of alignments in MEGA v. 6.06 using Muscle and ClustalW [26–28], phylogenetic analysis in MrBayes and RAxML [29,30], and synteny when the target gene was present on a contig of suitable size [25,31]. Synteny figures were constructed by hand, based on Ensembl's Region in Detail and NCBI's Genomic Region tools. When syntenic genes appeared to be missing for a species of interest, or when the genome browsers provided conflicting results, BLAST searches were carried out to determine if automated annotation tools had missed (or mislabelled) the gene. These regions were also visually inspected for large gaps or Ns that could result in a gene being dropped from the final assembly. Additional homologues were identified and validated through this suite of analyses and a final collection of sequences was chosen to provide key phylogenetic positions, with priority given to sequences of high quality contained on large contigs.

**Figure 1.**
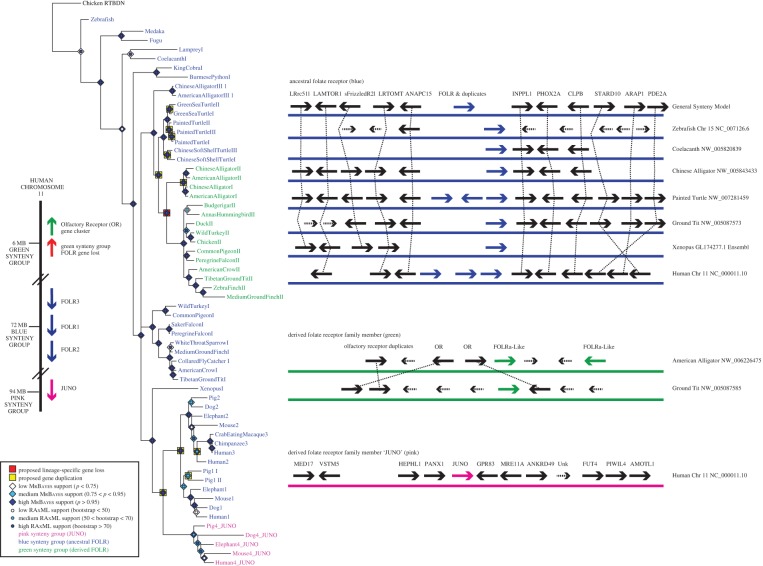
Phylogeny and synteny of the folate receptor family in vertebrates outlining three synteny groups: the ancestral folate receptor (blue), the derived folate receptor (green) and Juno (pink). Juno is only present in mammals. The phylogenetic tree was constructed using Bayesian and ML methods and support for each node is presented for each method as described in the legend. The Bayesian phylogeny was constructed following Jmodeltest2's [32] AICc-based recommendation of partitioned codon positions with the following models: GTR+G (position 1), HKY+G (position 2) and GTR+I+G (position 1). RAxML was run under the default model (GTR+CAT) partitioned by codon position with 1000 bootstraps. Syntenic relationships are shown with solid arrows connected by dotted lines. Non-syntenic genes are drawn as smaller, dotted arrows. Chromosome colour is representative of synteny group on the phylogeny.

The phylogeny and synteny presented here for the folate receptor family (figure 1) support the presence of a single ancestral folate receptor of the blue synteny group (generally adjacent to ANAPC15 and/or INPPL1) for ray-finned fish, lobe-finned fish and snakes, prior to lineage-specific duplications and losses which resulted in one Folr (currently listed as Folr1 on NCBI) in alligators, birds and *Xenopus*, two to three Folr genes in turtles, and three in mammals. All members of the blue synteny group have been maintained in this syntenic gene cluster. Two independent duplications with genomic translocation have also occurred, producing additional synteny groups. The green synteny group is known only from archosaurs, with two translocated Folr genes in alligators and one in birds. Juno, the pink synteny group, appears to have split from the ancestral (blue group) folate receptor early in mammalian evolution and has been maintained in its new chromosomal location in both marsupials and placental mammals.

In opossum (*Monodelphis domestica*), Juno's interaction with Izumo1 has been shown to be conserved [2], and its presence in the genome is supported through both synteny (electronic supplementary material, figure S1) and phylogeny. Due to genome quality, the evolutionary history of this gene family could not be fully assessed in platypus (*Ornithorhynchus anatinus*), although flanking genes from the pink syntenic group are present, while Juno appears absent (scaffold NW_001794203), and at least one partial Folr gene is present in the platypus genome (XM_001513334).

**Figure 2.**
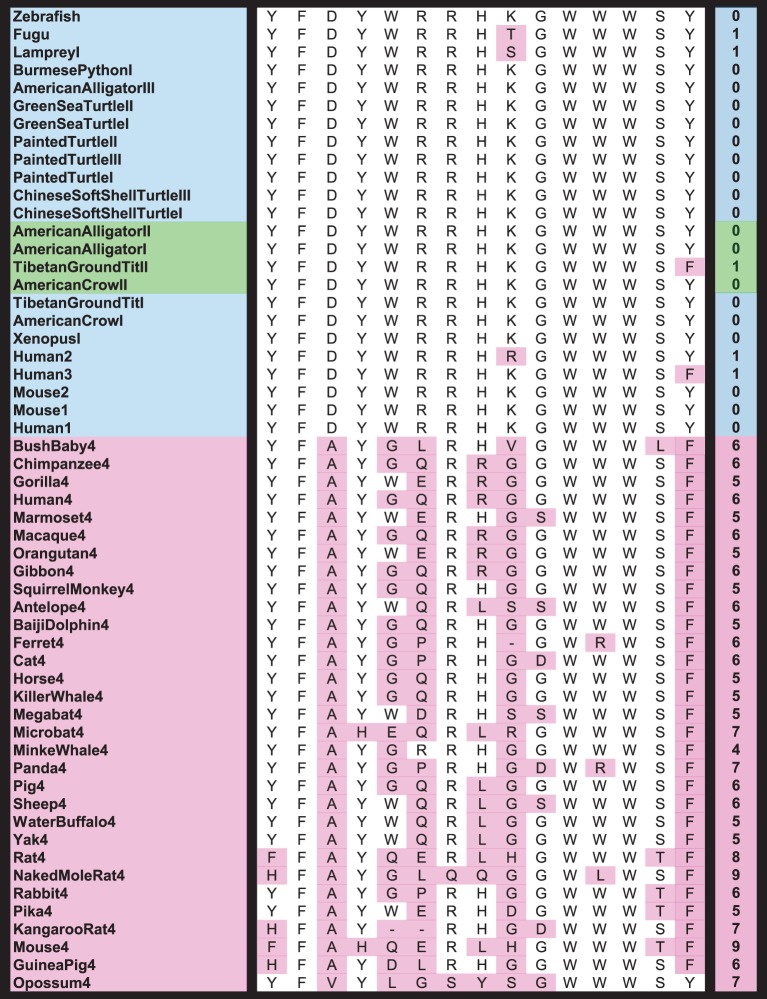
Sites 53, 55, 74, 78, 102, 103, 106, 135–138, 140, 171, 174 and 175 from the mature human Juno protein aligned with representative members from both the extended folate family (blue and green synteny groups) and mammalian Juno (pink synteny group) support the conservation of Juno in only Mammalia. These 15 sites are essential for folate binding in other Folr proteins, while Juno is known to be unable to bind folate. Derived sites are highlighted in pink, and the total number of substitutions present in each species is displayed in the far right column.

The absence of Juno from non-mammalian genomes is supported through phylogeny (figure 1), synteny analysis and examination of sites essential for folate binding (figure 2). Synteny of Juno's flanking genes is conserved throughout this region across most vertebrates, but Juno is only present in mammals (electronic supplementary material, figure S1); in addition to the standard BLAST searches on NCBI, each syntenic region in tetrapods and fish was downloaded and individually examined with BLAST+ using TBLASTN, TBLASTX and BLASTP to confirm Juno's absence [33].

Fifteen sites known to be essential for folate binding [2,21,22] were also examined *in silico*. Although a few sequences belonging to the blue and green synteny groups have one substitution among these 15 essential sites, the vast majority of non-Juno sequences show full conservation across all 15 sites in tetrapods and fish (figure 2). Alternatively, all Juno sequences examined have between four and nine substitutions across these sites. Four of these substitutions at essential folate-binding sites may have been crucial for Juno's neofunctionalization from the ancestral Folr gene, since they appear in all mammalian Juno genes examined with the exception of two species which each have the ancestral amino acid at one of the four sites. These patterns support the hypothesis that all Folr family members of the blue and green synteny groups (figure 1) possess the ability to bind folate and that Juno's loss of functional folate-binding predates the divergence of marsupial and eutherian mammals.

### 2.2 Izumo family evolution

Orthologous sequences for Izumo1, Izumo2, Izumo3 and Izumo4 were identified using the protocol outlined above for the Folr family. Additionally, Izumo family members share an approximately 150 amino acid ‘Izumo domain’ near their N-terminus, and all Izumo1 sequences possess an immunoglobulin-like domain [1,14]. These domains also aided in the validation of newly discovered Izumo proteins.

**Figure 3.**
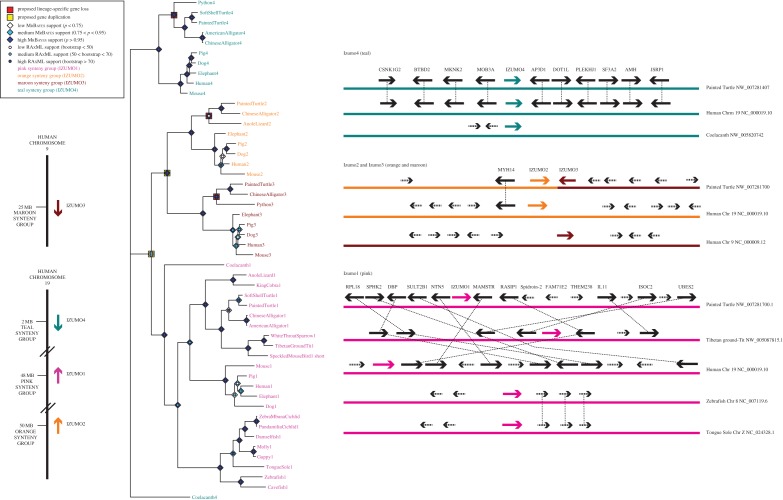
Phylogeny and synteny analysis of the extended Izumo family including the newly discovered, non-mammalian family members. Tree construction, coloration and synteny-mapping were carried out as in figure 1 (except *GTR*+*G* was used for all three codon positions during Bayesian reconstruction based on the AICc value in Jmodeltest2). Izumo1 is present in fish, reptiles, birds and mammals, Izumo4 is found in fish, mammals and reptiles, and Izumo2 and Izumo3 are only present in mammals and reptiles. These analyses support an ancient origin for the Izumo family.

The synteny and phylogeny (figure 3) support the discovery of non-mammalian members of all four Izumo paralogues, thereby placing the origin of this gene family 200–250 million years earlier than previous reports. Izumo2 and Izumo3, which are adjacent on reptilian contigs, appear to share a common ancestor with Izumo1, while Izumo4 is placed most distantly. This result is not surprising in light of Izumo4's absent transmembrane domain and expanded expression pattern [14].

In searching for Izumo genes, an interesting pattern emerged: despite the increasing number of assembled and annotated avian genomes (approx. 60 on NCBI), Izumo1 was difficult to identify in all but the Tibetan ground tit (*Pseudopodoces humilis*—the only bird to have a full Izumo1 gene identified). The other Izumo paralogues were not detected in birds, despite conservation in mammals and reptiles. Additional avian Izumo1 sequences were discovered through SRA BLAST, and the short, exonic sequences identified are reported (electronic supplementary material, table S1).

One common bias in sequence data is underrepresentation of GC- and AT-rich genomic regions [34]. Izumo1's GC content is approximately 45–61% in fish and mammals, approximately 55–68% in reptiles and 71% for Tibetan ground tit. This high GC content within the CDS of the ground tit is also present in the surrounding genomic regions (65% GC, compared to 55% and 57% for human and turtle, respectively). Syntenic genes, including RASIP1 and ISOC2, are also absent from all avian genomes except the ground tit. These findings might explain the absence of Izumo1 sequences for many birds. Non-avian Izumo paralogues share fairly consistent GC content within a given species, a pattern that (if maintained in birds) could suggest that Izumo2–4 are not lost, but simply hidden due to sequencing bias (figure 3). Alternatively, these paralogues may have been removed during the massive avian-specific loss of ancestral genes originally identified in chicken [35].

### 2.3 Positive selection and coevolution in Izumo1 and Juno

**Figure 4.**
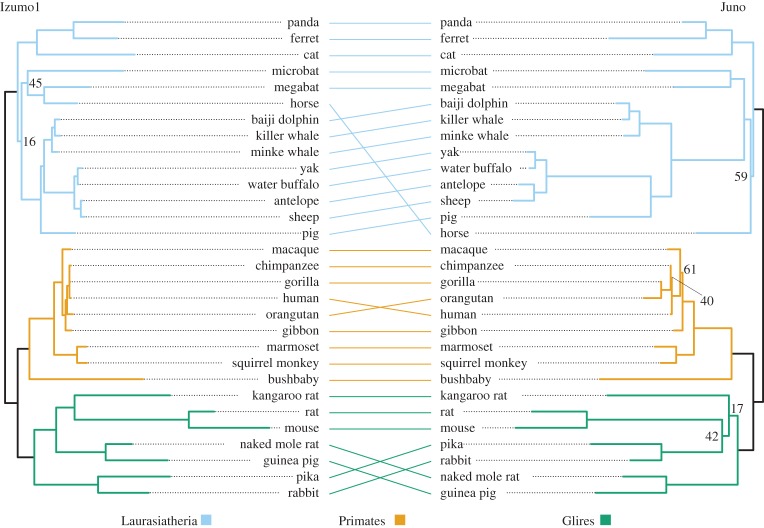
Mirrored gene trees for Izumo1 and Juno containing the 30 common mammals used in PAML (table 1) and Mirrortree (table 2) analyses. Trees were constructed using RAxML under the default model (*GTR*+*CAT*) partitioned by codon position with 1000 bootstraps. Bootstrap support values below 70 are indicated. This figure was constructed using the APE [38] and phytools [39] packages in R.

For both Izumo1 and Juno, phylogenetic analysis by maximum likelihood (PAML) [36,37] identified signals of positive selection across 30 common mammals with full, annotated genomes and good synteny in the target regions (table 1 and figure 4; see electronic supplementary material, table S1c for accession numbers). These results support those reported for one of the best-studied invertebrate sperm–egg partnerships, the lysin–VERL system of marine abalone. Both lysin on the sperm and VERL, its receptor on the egg, evolve under adaptive evolution [10,40]. Non-mammalian Izumo1 in birds, reptiles and fish appears to be evolving under purifying selection (table 1).

**Table 1.** Positive selection is acting on both members of the essential sperm–egg binding pair (Izumo1/Juno), but is confined to different lineages. PAML and Bayes empirical Bayes (BEB) results for clade-based selection tests. Likelihood ratio test values are presented in columns M7 versus M8 and M8 versus M8a. M7 allows *ω* to vary between 0 and 1, while M8 adds an additional parameter whereby *ω* can exceed 1; M8a (M7 with *ω*=1) is the null hypothesis for M8 [36]. The total number of BEB sites with probability greater than 0.5 are reported before the semi-colon, followed by those sites identified as having *p*-values of 0.95 or better [37]. BEB sites were only produced for clades evolving under positive selection.

gene	group	*n*	M7 versus M8	M8 versus M8a	BEB sites
Juno	mammals	30	12.87***	11.32***	5; 115L***
Juno	Glires	7	1.39	0	n.a.
Juno	Laurasiatheria	14	1.05	0.74	n.a.
Juno	Primates	9	11.17***	7.83***	8; 111T*, 113L^**^, 123I*
Izumo1	mammals	30	65.03***	43.32***	101; 28P*, 167P***, 261S^**^, 274A*, 276T*, 303M* 307E^**^, 308L***, 310T***, 390E^**^, 391T*, 400R*, 403R***
Izumo1	Glires	7	2.72	0.99	n.a.
Izumo1	Laurasiatheria	14	53.77***	44.68***	65; 28P^**^, 118H*, 167K***, 173E*, 193I*, 274Q*, 277T*, 278A*, 298V^**^, 302-*, 303P*, 353-*, 354T*, 382-*, 385-^**^
Izumo1	Primates	9	0.68	0.37	n.a.
Izumo1	non-mammals	18	0.50	0.30	n.a.

*p≥0.95, ^**^p≥0.975, ***p≥0.99.

**Table 2.** Mirrortree Web Server [42] identifies lineage-specific signals of coevolution between mammalian Izumo1 and Juno. Correlation values were generated using both Newick trees created in RAxML (figure 4), and multiple sequence alignments (MSA) created with Muscle. Italicized correlation values lie above the Mirrortree correlational cut-off of 0.800 and are suggestive of coevolution between Izumo1 and Juno within that group.

		correlation		correlation	
group	*n*	value from trees	*p*-value	value from MSA	*p*-value
mammals	30	0.720	<0.000001	*0*.*817*	<0.000001
Glires	7	*0*.*866*	<0.000001	0.690	<0.000001
Laurasiatheria	14	0.669	<0.000001	0.775	<0.000001
Primates	9	*0*.*990*	<0.000001	*0*.*989*	<0.000001

Previous in-depth analysis of molecular evolution within the Izumo family identified that the positive selection driving mammalian adaptive evolution for Izumo1 is localized to the laurasiatherian mammals, being absent from both Glires and Primates [13]. To determine if this pattern is also present in Juno, suggestive of coevolution between the binding partners, each clade was tested independently for both Izumo1 and Juno (table 1). These analyses have never been performed on Juno and are more robust than those previously carried out for Izumo1 in both species number and sequence coverage. Izumo1's signal of selection within laurasiatherians was present once more, and Juno's mammalian signal of positive selection appears to be driven by the Primates alone (table 1).

An additional technique used to investigate protein coevolution, called Mirrortree, compares two gene trees (e.g. a ligand and its receptor) to detect similarities greater than those expected under a standard molecular clock. Strong correlations (defined as more than 0.800) are suggestive of coordinated changes in both genes, resulting from similar evolutionary forces [41]. Mirrortree was used to examine the same 30 mammalian species used in the PAML analysis for coevolutionary signals between the gene trees of Izumo1 and Juno.

Correlation values between Izumo1 and Juno were generated through two separate analyses on the Mirrortree Web Server [42]: (i) using the ‘From trees’ option and (ii) using the ‘From multiple alignments’ option (table 2, figure 4). The ‘From trees’ approach analysed the same RAxML trees used for PAML analyses (figure 4), while the ‘From multiple alignments’ method examined a Clustal NJ tree produced on the Web Server from provided Izumo1 and Juno protein alignments [42]. Both analyses identified a robust signal of coevolution between Izumo1 and Juno within Primates (table 2). The ‘From trees’ approach also identified a signal of coevolution within the Glires, but not across all 30 mammals or within the Laurasiatheria (table 2). The ‘From multiple alignments’ method did not identify a signal in Glires or Laurasiatheria, but did show evidence of coevolution across all 30 mammals analysed (table 2).

These results support the hypothesis that Juno and Izumo1 are experiencing coordinated evolution within the mammals. The strength of the Mirrortree signal appears to vary by clade and by method of analysis. The discrepancies seen between the two methods could be explained by tree support. For example, low bootstrap support within a portion of the Laurasiatheria (figure 4) might factor into the non-significant signal of coevolution detected for that clade. The Izumo1 clade containing horse (*Equus caballus*) has a bootstrap support value of 45 (figure 4), and the horse phylogenetic position is one of the least correlated across the analysed mammals. Additionally, the ‘From trees’ approach used trees built from nucleotide alignments, while the ‘From multiple alignments’ method requested protein alignments, which it used to construct each phylogenetic tree. Analysing trees built using nucleotides versus proteins could also affect the signals identified through Mirrortree.

## 3. Discussion and conclusion

Here, I describe the evolutionary history of Juno and Izumo1 in the context of their gene families. In doing so, new Izumo genes in tetrapods and fish with potential roles in reproduction are described for the first time, and the mammalian origin of Juno is elucidated. The discovery of disparate evolutionary origins for these proteins raises a number of future research questions. What is the function of Izumo1 outside of Mammalia? How did the interaction between Izumo1 and the ancestral folate receptor first arise? Since sperm, eggs and the modes of fertilization vary across the vertebrate phylogeny, whether non-mammalian Izumo genes possess reproductive functionality is currently uncertain, but merits further investigation. Targeted examination of expression patterns for these genes across the phylogeny and throughout the vertebrate body is one potentially fruitful approach to address these questions.

Through bioinformatic analyses, I present evidence that the first essential sperm–egg binding pair discovered in any organism is evolving under positive selection. I demonstrate that different clades have driven the positive selection identified in each binding partner, and that the mammalian conservation of Izumo1–Juno binding ability and their essential function in sperm–egg recognition have probably resulted in coevolution between these two proteins. These results support the long-standing hypotheses that rapid evolution and positive selection observed in reproductive proteins are often driven by coevolution between male and female proteins, either as an arms race (through sexual conflict) or through adaptations to species-specific gamete recognition systems that are key to avoiding hybridization. For example, positive selection and coevolution between lysin and VERL of marine abalone are believed to have resulted from one or both of these processes [10,43].

It is unlikely that Izumo1 and Juno play major roles in species-specific gamete recognition for two reasons. The first is that Juno localizes on the egg membrane. This membrane sits interior to the zona pellucida (ZP), a major barrier in preventing hetero-specific sperm from binding to eggs in mammals [44]. The second is the recent finding that cross-species Izumo1–Juno binding is possible between human Izumo1 and Juno on the Syrian golden hamster (*Mesocricetus auratus*) egg following removal of the ZP. In this species pair, it appears that Juno can bind hetero-specific Izumo1; however, this trend does not appear to be consistent across mammals [3]. The same manuscript reports that human Izumo1 does not bind to mouse Juno, suggesting that some level of species recognition might be carried out between Izumo1 and Juno after sperm penetrates the ZP [3].

The possibility that Izumo1 and Juno are coevolving under the pressures of sexual conflict is also an interesting one, which would probably be best studied in a small clade of species with varied levels of sperm competition. A recent study using this model identified positive selection and coevolution between Izumo1 on sperm and CD9 on the egg across the *Mus* genus [7]. The influence of sexual selection on the evolution of a protein pair can be examined by comparing lineage-specific *ω* values (an indicator of selective pressure) to a proxy of sexual selection (e.g. relative testes mass). This method produced negative results for CD9 and Izumo1, but could be applied to Juno and Izumo1 in the future [7].

While it is clear that selection analyses conducted across distantly related species can uncover signals of positive selection at high taxonomic levels (e.g. Mammalia), it is important to note that they often overlook lineage-specific or recent bouts of selection, and can be biased based on the sequenced taxa that are currently available [45,46]. For example, Izumo1 is reportedly evolving under positive selection in *Mus*, despite appearing to be under the influence of purifying selection in Glires [7]. In other instances, the coarse signals identified by analysing previously sequenced species remain unchanged with the addition of new closely related species. When Izumo1 was first studied within Primates, purifying selection was identified using sequence data for just five species from online databases [13]. Recently, both CD9 and Izumo1 were sequenced and analysed in 12 Primates, and neither gene was found to be evolving adaptively in these groups [47]. If there are lineage-specific bouts of positive selection acting on Izumo1 within Primates, they are probably hidden at the genus level, as seen in *Mus*.

Although Izumo1 and CD9 do not appear to be evolving under positive selection in the Primates, they again display signals of coevolution [47]. Izumo1 and CD9 are not known to interact directly, but the coevolutionary signals identified in *Mus* and Primates are suggestive of either functional associations or interaction at the molecular level [7,47]. A similar conclusion can be drawn from the signal of coevolution identified between Izumo1 and Juno in the current paper. Since the Izumo1–Juno signal of coevolution has already been validated through molecular data describing the essential sperm–egg binding behaviour of these proteins, my results lend support to methods such as Mirrortree for candidate gene screening and potential identification of interacting proteins using bioinformatics [2,48]. Understanding the evolutionary histories of gene families and examining patterns of coevolution can help inform functional studies in humans, model and non-model organisms.

## Data accessibility

Raw data as well as supplementary tables and figures are available from the Dryad Digital Repository (http://dx.doi.org/10.5061/dryad.gh208).
